# Integrator Drift Compensation of Magnetic Flux Transducers by Feed-Forward Correction †

**DOI:** 10.3390/s19245455

**Published:** 2019-12-11

**Authors:** Maria Amodeo, Pasquale Arpaia, Marco Buzio

**Affiliations:** 1 Department of Electronics and Telecommunications (DET), Polytechnic University of Turin, 10138 Turin, Italy; maria.amodeo@cern.ch; 2 Instrumentation and Measurement Laboratory for Particle Accelerator Laboratory (IMPALab), Department of Electrical Engineering and Information Technology (DIETI), University of Naples Federico II, 80100 Naples, Italy; 3 Technology Department, CERN, 1217 Geneva, Switzerland; Marco.Buzio@cern.ch

**Keywords:** magnetic measurements, magnetic sensor, Nuclear Magnetic Resonance (NMR), magnets, particle accelerator, integrator drift

## Abstract

Integrator drift is a problem strongly felt in different measurement fields, often detrimental even for short-term applications. An analytical method for modelling and feed-forward correcting drift in magnetic flux measurements was developed analytically and tested experimentally. A case study is reported on the proof of principle as a novel kind of quasi-DC field marker of the 5-ppm Nuclear Magnetic Resonance (NMR) transducer Metrolab PT2026, applied to the Extra Low ENergy Antiproton (ELENA) ring and the Proton Synchrotron Booster (PSB) at CERN. In some particle accelerators, such as in ELENA, the resulting feed-forward correction guarantees 1 μT field stability over 120-s long magnetic cycle on a plateau of 50 mT, reducing by three orders of magnitude the field error caused by the integrator drift with respect to the state of the art.

## 1. Introduction

The problem of voltage integrator drift is strongly felt in different domains where real-time measurements are needed, from inertial to magnetic sensors. In general, drift is due to a systematic offset or random noise in the measured voltage, in particular the well-known 1/f noise [1]. Different physical mechanisms causing an offset include thermoelectric voltages in the acquisition chain, rectification of electromagnetic interference by non-linear circuit elements, and imbalance of discrete and integrated circuital components.

In the domain of magnetic field measurement, the problem of drift correction arises notably in Tokamaks. These are experimental devices designed to contain a thermonuclear plasma by means of a combination of cyclically pulsating magnetic fields to be known within 1% throughout the duration of a single discharge. The magnetic field is measured by dividing the flux, obtained by integrating the output of a stationary induction coil, by the area of the coil [2]. For machines such as the experimental reactor ITER currently in construction at Cadarache, France, drift correction will be especially important, due to the 1000-s nominal discharge duration [3]. In the analog integrators of KSTAR (Daejeon, South Korea), the offset was obtained from the average of the measured integrator input signal during the dead time between discharges, when the coil output is identically null [4]. The offset was subsequently subtracted from the input signal via a DAC connected in series. This technique relies on the a-priori assumption that the magnetic field is constant at least for 30 s. Moreover, it cannot take into account any possible fluctuations of the offset during a long discharge. A more recent FPGA-based version of the integrators uses instead the well-known chopping technique, which is based on inverting the input signal at high frequency, so that any offset generated within the integrator itself is alternately added and subtracted [5]. After integration, the original polarity is restored by inverting the chopping process, and the final result is filtered to average out the offset. This method suffers from several drawbacks, such as the injection of high-frequency perturbations from the chopping, or the loss of bandwidth plus the phase lag due to the necessary filtering. In addition, any offset due to sources external to the integrator itself, such as thermocouple effects in the coil wiring, or semiconductor effects in the pre-amplifier stage, remains embedded in the signal. An analogous concept, with comparable disadvantages, is implemented in EAST (Hefei, China), where the measurement is obtained by switching at high frequency between two integrators in parallel, one short-circuited to measure the offset while the other is connected to the coil [6]. Also in this case, only the internal sources of offset are taken into account, while the external ones are ignored.

The inductive (fluxmetric) measurement of magnetic field is also used extensively in the domain of particle accelerator magnets. The most common case is the rotating-coil measurement of a static field, carried out during the acceptance tests. In this case, correction of integrator drift is facilitated by the very short integration times, typically one second or less, and by the periodicity of the measured flux as a function of the coil rotation angle [7]. Often the magnets are operated with fast magnetic cycles, so that measurements with a fixed induction coil are useful to capture dynamic effects. In certain cases, these measurements are needed in real time, for example to feed back magnetic field information to various machine control systems. The requirements for these applications may be very demanding, up to 100 ppm or better in terms of relative accuracy, and hundreds of kHz of bandwidth, which makes the drift correction techniques described above hardly suitable.

In this paper, a feed-forward method to model and correct integrator drift in real time under these conditions is discussed. The method is designed in particular for long, quasi-static field plateaus, frequent in accelerator operation. As an example of future application, the method is applied to two machines in operation at CERN, the Extra Low ENergy Antiproton (ELENA) [8] ring and the Proton Synchrotron Booster (PSB) [9]. Section 2 provides the background on the measurement to be carried out. Section 3 focuses on the measurement model and the proposed feed-forward drift correction method. Section 4 describes the case study, showing the experimental results obtained with a DC field source and, finally, the conclusions are drawn in Section 5.

## 2. State-Of-The-Art Drift Correction for Fixed Induction Coils in Accelerator Magnets

This work is concerned with magnetic measurements for synchrotrons, a class of ring-shaped particle accelerators where the momentum of the circulating particles varies in proportion to the magnetic fields that guide and control the beam. In these machines, precise knowledge of the integrated field of the dipoles that bend the beam trajectory around the ring is essential for transversal and longitudinal beam control [10]. The instantaneous value of the magnetic field is used by the RF accelerating cavities, the main dipole power supplies, as well as by certain beam diagnostics and by the operation team in the control room. Synchrotrons are used not only for high-energy physics research, but also for medical purposes, as in the case of the *National Centre for Oncological Hadrontherapy* (CNAO, Pavia, Italy), a hospital center dedicated to the treatment of tumors by hadrontherapy [11]. This therapy may be useful for treating radio-resistant or deep-seated, inoperable tumors. Targeting the tumor while, at the same time, sparing the adjacent tissues requires the beam position to be controlled with a precision of about 0.1 mm, which in turn implies that bending magnetic field must be known within an accuracy of 100μT [12].

In principle, the field produced by electromagnets can be derived mathematically as a function of the excitation current. In some special cases, such as the conductor-dominated, very high-field (8.4 T) LHC superconducting dipoles, the field can be predicted accurately enough by means of semi-empirical mathematical models [13]. However, in the more common case of iron-dominated magnets, precise prediction of non-linear and dynamic effects is beyond the current state of the art. While a number of well-established techniques exist to improve reproducibility of the magnets (e.g., by pre-cycling [14]) and to stabilize the field on flat-tops (e.g., by applying current overshoots [15]), these are often too costly in terms of lost beam time. In such cases, feedback control based on magnetic field measurements is necessary.

At CERN, five so-called “*B-train*” systems are used to measure in real-time the field of a reference magnet, which can be either one of those in the ring or an additional unit, installed in a separate facility and excited in series with the ring [16]. The measurement principle of these systems is based on the fluxmetric method, derived from Faraday’s law:

(1) $$B\left(t\right)=B_{0}+\frac{1}{A_{c}}\int_{t_{0}}^{t}V_{c}\left(\tau \right)\;d\tau ,$$

where *B* is the average field in the bending dipoles, $B_{0}=B\left(t_{0}\right)$ is the integration constant and $V_{c}$ is the voltage output of an induction coil of effective area $A_{c}$. The time integral of the coil voltage has the physical dimensions of magnetic flux. Induction coils have important advantages: they can be linear up to the range of hundreds of kHz, are easy to calibrate accurately, and are able to measure the average field of very long magnets, up to several metres. Commonly, the order of magnitude of $A_{c}$ is ∼1 m${}^{2}$, so that with peak field ramp rates of a few T/s the maximum output voltage does not exceed a few volts.

In the systems currently in operation at CERN, the integration constant $B_{0}$ is usually a pre-defined, fixed value. The starting time of the integration $t_{0}$ is triggered by an additional sensor called a *dynamic field marker*, which emits a TTL pulse whenever the field crosses the given threshold $B_{0}$. The Metrolab PT2025 Nuclear Magnetic Resonance (NMR) transducers [17] are often used in continuous-wave mode for this purpose, as discussed in detail in [18]. These transducers are extremely accurate, although they are somewhat limited by the requirement of a minimum field ramp rate, of the order of 0.2 T/s, in order to generate a reliable trigger. This has never been an issue so far, since the integration always starts during the fast field up-ramp preceding the injection of the beam in the ring. In the following analysis, the NMR measurement is assumed as reference without any uncertainty.

The integration proceeds then for the duration of a magnetic cycle as a whole, typically a few seconds, and is restarted on each cycle. This prevents effectively the build-up of integrator drift from cycle to cycle.

Measurements of pulsed fields with fixed induction coils are very common both off line, for acceptance and qualifications tests, and on line, for B-train systems. In principle, drift correction is similar in both cases: the DC offset voltage is first estimated by averaging the voltage at the input of the integrator over a time interval when no excitation is applied to the magnet, and then is subtracted from the input signal. The offset should be estimated as often as possible, because it is not always stable.

In three of the B-train systems currently in operation at CERN (Proton Synchrotron, Proton Synchrotron Booster, and Low Energy Ion Ring), the estimation is carried out during special machine cycles, the so-called *zero cycles*. During them, there is no beam and the magnet power supplies follow specific maintenance cycles, that may or may not include current plateaus. The presence and the timing of a constant-field interval are not always guaranteed during a zero cycle, therefore the integrator input is short-circuited in order to estimate the offset. As in the literature examples cited above, the obtained correction is only partial, since it takes into account only the contribution of the internal electronics and neglects external sources. In addition, zero cycles consume valuable beam time, and therefore machine operators tend to make them available only sporadically, or preferably not at all.

The drift observed to date in the daily operation of CERN B-train systems is typically of the order of 10 to 100 μT/s, which is acceptable for short cycles. However, future high-luminosity operation of the LHC will demand higher beam intensities, more accurate magnetic field control and less zero cycles. Moreover, in certain machines the cycle length, and therefore the uninterrupted integration duration, is planned to increase considerably. The most stringent requirements are an accuracy of 1 μT, i.e., 20 ppm on 50 mT plateaus lasting up to 120 s for the ELENA ring, where zero cycles are not available at all. It should be emphasized that a rigorously constant field cannot be simply achieved by exciting the magnet with a constant current in open loop, due to various kinds of perturbation such as current ripple, the decay of eddy currents in the magnet’s iron yoke, and thermally-induced drifts. For these reasons, a better correction method is necessary. The method proposed below is implemented in a new generation of B-train systems which is being developed and tested at CERN, in the context of a general consolidation project, with the goal to improve their performance and guarantee their long-term maintainability.

## 3. Proposed Modelling and Correction of Integrator Drift

The correction method to be implemented in the new real-time measurement systems is based two main concepts:

- replacing the preset integration constant $B_{0}$ in Equation (1) by a field measurement
- using two consecutive field marker measurements to estimate the voltage offset, which is henceforth subtracted from the voltage signal

For example, a Hall probe able to provide a readout on-demand, rather than at an uncontrolled time such as in the case of a field marker, is used at the HIT hadronteraphy center, Heidelberg [19]. The transducer chosen at CERN is the recently released Metrolab PT 2026, which combines the very high (∼5 ppm) absolute accuracy of NMR with fast pulsed-mode operation [20]. This instrument, unlike the earlier continuous-wave version PT2025, is limited instead by high field ramp rates, which must be less than 0.5 mT/s at the level of 50 mT. The two instruments therefore complement each other to take the field marker role under fast-ramping and quasi-static conditions respectively.

### 3.1. Analytical Model

**Figure 1:**
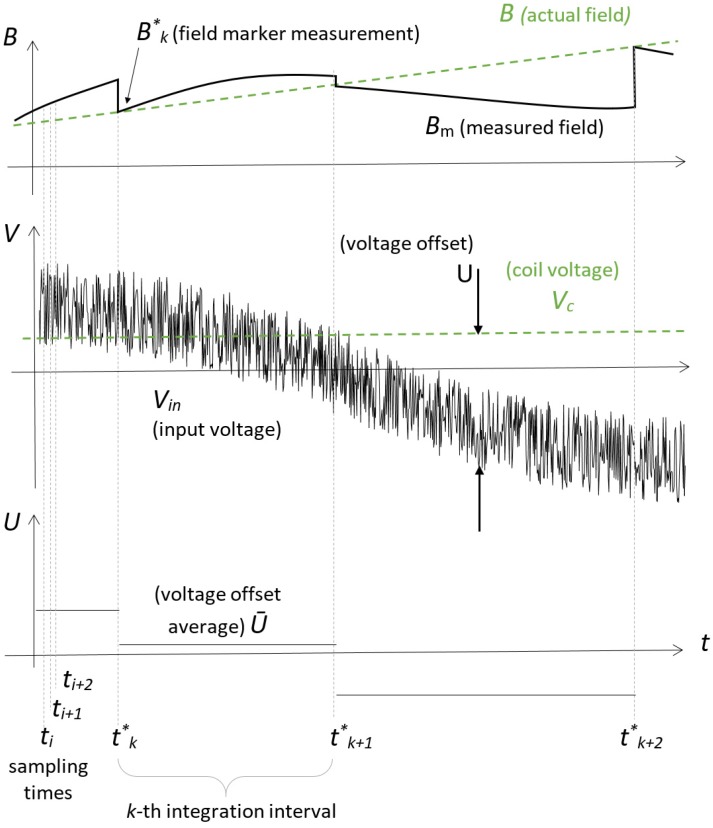
Actual and measured magnetic field (**top**), voltage offset (**middle**) and average voltage offset over consecutive integration intervals (**bottom**). The curves represent the ideal case of a field varying at constant rate, causing a constant nonzero coil voltage that is superposed to noise and a slowly variable offset. The typical sampling period of the discrete waveforms is three to four orders of magnitude shorter than the integration intervals.

The measurement model is based on segmenting the time axis into consecutive integration intervals, denoted by the index *k*, as shown in Figure 1.

At the beginning of each interval the field is measured by the NMR transducer, triggered via software, then the variation is obtained continuously by integrating the output of the sensing coil. The star symbol denotes all quantities evaluated at this time. In the following, for simplicity, integration is represented in the continuous time domain; in reality, however, the coil voltage is sampled by an ADC at the rate of 2 MS/s and all the subsequent processing is carried out in the discrete time domain by an FPGA. The NMR sensor is sensitive to the modulus of the magnetic field vector; however, this is practically equal to the vertical component measured by the coil, due to the very high uniformity of the field in the dipole (better than 50 ppm). The magnetic field is given by:

(2) $$B\left(t\right)=B_{k}^{*}+\frac{1}{A_{c}}\int_{t_{k}^{*}}^{t}V_{c}\left(\tau \right)\;d\tau ,\;\;\;\;t_{k}^{*}\leq t<t_{k+1}^{*},$$

where $B_{k}^{*}=B\left(t_{k}^{*}\right)$ is the NMR measurement at the start of the interval. All integration intervals shall have the same nominal duration $T^{*}$. Each $t_{k}^{*}$ is affected by uncertainty due to the software triggering and the latency inherent in the teslameter itself (see Section 4.1), therefore the actual duration of each interval may be slightly different:

(3) $$T_{k}^{*}=t_{k+1}^{*}-t_{k}^{*}\approx T^{*}.$$

The voltage $V_{\mathrm{in}}$ at the input of the integrator is the sum of two contributions: the output voltage of the coil and the offset voltage *U*, which is usually in the range of tens to hundreds of microvolts:

(4) $$V_{\mathrm{in}}\left(t\right)=V_{c}\left(t\right)+U\left(t\right).$$

In the absence of any correction, the measured field $B_{m}$ can therefore be expressed according to Equation (5):

(5) $$B_{m}\left(t\right)=B_{k}^{*}+\frac{1}{A_{c}}\int_{t_{k}^{*}}^{t}V_{\mathrm{in}}\left(\tau \right)\;d\tau .$$

The measured and actual field are related by:

(6) $$B_{m}\left(t\right)=B\left(t\right)+\frac{1}{A_{c}}\int_{t_{k}^{*}}^{t}U\left(\tau \right)\;d\tau .$$

### 3.2. Voltage Offset Model

On time scales of a few minutes to a few hours, the voltage offset is typically observed to fluctuate randomly around a nonzero systematic value. As a useful first approximation, it will be assumed that during the *k*-th integration interval the offset can be considered equal to its mean value ${\bar{U}}_{k}$, as shown in Figure 1:

(7) $${\bar{U}}_{k}=\frac{1}{T^{*}}\int_{t_{k}^{*}}^{t_{k+1}^{*}}U\left(\tau \right)\;d\tau ,\;\;\;\;t_{k}^{*}\leq t<t_{k+1}^{*}.$$

In the operational B-train systems, the mean offset can be characterized experimentally by taking the interval duration $T^{*}$ equal to the cycle period and evaluating the RMS average over $N_{c}$ consecutive cycles (see Section 4.2):

(8) $$U_{\mathrm{RMS}}=\sqrt{\frac{1}{N_{c}}\sum_{k=1}^{N_{c}}{\bar{U}}_{k}^{2}}.$$

The observed fluctuations of the offset with time can be characterized in a simple manner by taking the RMS average of its discretized time derivative:

(9) $${\left(\frac{d\bar{U}}{dt}\right)}_{\mathrm{RMS}}=\sqrt{\frac{1}{N_{c}-1}\sum_{k=2}^{N_{c}}\frac{{\left({\bar{U}}_{k}-{\bar{U}}_{k-1}\right)}^{2}}{T^{*2}}}.$$

### 3.3. Feed-Forward Offset Correction

The error accumulated during the *k*-th integration interval can be evaluated very precisely by the difference between the current field measurement and the NMR field marker readout that signals the beginning of the next interval:

(10) $$\Delta B_{k}^{*}=B_{m}\left(t_{k+1}^{*}\right)-B_{k+1}^{*}=B_{k}^{*}-B_{k+1}^{*}+\frac{1}{A_{c}}\int_{t_{k}^{*}}^{t_{k+1}^{*}}V_{\mathrm{in}}\left(\tau \right)\;d\tau .$$

If the system’s output was given directly by Equation (5), it would therefore jump abruptly by $-\Delta B_{k}^{*}$ at $t=t_{k+1}^{*}$, as shown in Figure 1. Such a discontinuity is undesirable, because it could affect the stability of the RF and magnet power supply control loops. For this reason, in practice the jump is smeared out linearly over a time of the order of 10 ms. Assuming that the accumulated error is due only to integrator drift, the average offset during a completed interval can be derived from Equation (6):

(11) $${\bar{U}}_{k}=\Delta B_{k}^{*}\frac{A_{c}}{T^{*}}.$$

This knowledge can be fed forward to the next integration interval, in order to attempt at least a partial correction of the measurement in real time. During the *k*-th interval, the offset $U_{k}$ can be expressed as:

(12) $$U_{k}\left(t\right)={\bar{U}}_{k-1}+\delta U_{k}\left(t\right),$$

where $\delta U_{k}$ represents the fluctuating component and, on average:

(13) $${\bar{U}}_{k}\left(t\right)={\bar{U}}_{k-1}+{\left(\frac{d\bar{U}}{dt}\right)}_{k}T^{*}.$$

Feeding forward the offset obtained in the previous cycle, the measured field Equation (5) can be expressed as:

(14) $$B_{m}\left(t\right)=B\left(t\right)+\frac{{\bar{U}}_{k-1}}{A_{c}}\left(t-t_{k}^{*}\right)+\frac{1}{A_{c}}\int_{t_{k}^{*}}^{t}\delta U_{k}\left(\tau \right)d\tau .$$

Finally, a corrected version of the field $B_{\mathrm{mc}}$, i.e., the final output of the algorithm, can be defined by:

(15) $$B_{\mathrm{mc}}\left(t\right)=B_{m}\left(t\right)-\frac{{\bar{U}}_{k-1}}{A_{c}}\left(t-t_{k}^{*}\right)=B_{k}^{*}+\frac{1}{A_{c}}\int_{t_{k}^{*}}^{t}V_{\mathrm{in}}\left(\tau \right)\;d\tau -\frac{{\bar{U}}_{k-1}}{A_{c}}\left(t-t_{k}^{*}\right).$$

From Equation (14) the corrected field is related to the actual field by:

(16) $$B_{\mathrm{mc}}\left(t\right)=B\left(t\right)+\frac{1}{A_{c}}\int_{t_{k}^{*}}^{t}\delta U_{k}\left(\tau \right)d\tau .$$

### 3.4. Uncertainty Analysis of Voltage Offset

In this subsection, the standard uncertainty, associated to the estimated voltage offset and the measured field, is assessed. First, the uncertainty of the offset computed at the end of each integration interval is estimated by uncertainty propagation in Equation (11):

(17) $$\frac{\sigma^{2}\left({\bar{U}}_{k}\right)}{{\bar{U}}_{k}^{2}}=\frac{\sigma^{2}\left(A_{c}\right)}{A_{c}^{2}}+\frac{\sigma^{2}\left(\Delta B_{k}^{*}\right)}{{\Delta B_{k}^{*}}^{2}}+\frac{\sigma^{2}\left(T^{*}\right)}{T^{*2}}.$$

In this expression, three contributions to the relative uncertainty are found:

- the coil surface area. This contribution arises mainly from the uncertainty of the coil calibration process and is usually very low, i.e., a few $10^{-4}$ [21] in relative terms. This represents a systematic error that affects equally all measurements obtained from the coil, although not those obtained from the NMR field marker. Additional random fluctuations due to thermal effects can be neglected, since laboratory systems are usually stabilized in temperature;
- the measured field difference Equation (10); this includes two contributions, (i) from the NMR measurements, in the range of a few $10^{-6}$ in relative terms and therefore negligible, and (ii) from the flux integration, dominated by the uncertainty of the coil surface and therefore also small (see Appendix A);
- the measured duration of the integration interval. Relative error can be in the percent range, as shown experimentally in Section 4.1.

It can be seen that the uncertainty of the estimated voltage offset is largely dominated by the effective duration of the integration interval Equation (3), which in turn depends upon the jitter of the individual measurements of the initial and final times, so that:

(18) $$\frac{\sigma^{2}\left({\bar{U}}_{k}\right)}{{\bar{U}}_{k}^{2}}\approx \frac{\sigma^{2}\left(T^{*}\right)}{T^{*2}}=2\frac{\sigma^{2}\left(t^{*}\right)}{T^{*2}}.$$

As a result, from Equation (13) the total uncertainty of the voltage offset during the *k*-th interval can be expressed as the sum of two contributions: (i) one Equation (18) arising from the previous interval estimation, and (ii) one due to the fluctuating component Equation (9), assessed in terms of its RMS average:

(19) $$\sigma^{2}\left({\bar{U}}_{k}\right)=\sigma^{2}\left({\bar{U}}_{k-1}\right)+\sigma^{2}\left({\left(\frac{d\bar{U}}{dt}\right)}_{k}T^{*}\right)=2\frac{\sigma^{2}\left(t^{*}\right)}{T^{*2}}{\bar{U}}_{k}^{2}+{\left(\frac{d\bar{U}}{dt}\right)}_{\mathrm{RMS}}^{2}T^{*2}.$$

This expression emphasizes the negative impact of integration intervals that are either too short, thus causing a larger error in the estimation of the past mean offset, or too long, leaving more time for the random fluctuations to add up. An objective criterion for the choice of the optimal $T^{*}$ can be obtained by differentiating Equation (19) to seek its minimum $T_{\mathrm{opt}}^{*}$:

(20) $$T_{\mathrm{opt}}^{*}=\sqrt{\frac{\sigma \left(t^{*}\right)U_{\mathrm{RMS}}}{{\left(\frac{d\bar{U}}{dt}\right)}_{\mathrm{RMS}}}}.$$

### 3.5. Uncertainty Analysis of the Measured Field

The uncertainty of the corrected field can be obtained as a function of time by error propagation in Equation (15). By neglecting the contributions of the NMR measurement, the coil area and the flux integration, as done in the previous subsection, the following expression is obtained:

(21) $$\sigma^{2}\left(B_{\mathrm{mc}}\left(t\right)\right)\approx \frac{{\bar{U}}_{k-1}^{2}}{A_{c}^{2}}\frac{\sigma^{2}\left({\bar{U}}_{k-1}\right)}{{\bar{U}}_{k-1}^{2}}{\left(t-t_{k}^{*}\right)}^{2}+\frac{{\bar{U}}_{k-1}^{2}}{A_{c}^{2}}\sigma^{2}\left(t^{*}\right).$$

The measurement uncertainty, as it may be expected, grows with time due to the increasing impact of the uncertainty on the offset on integrator drift, so that at the end of the integration interval, by substituting Equation (18):

(22) $$\sigma^{2}\left(B_{\mathrm{mc}}\left(t_{k+1}^{*}\right)\right)\approx 3\frac{{\bar{U}}_{k-1}^{2}}{A_{c}^{2}}\sigma^{2}\left(t^{*}\right).$$

Finally, from Equation (16), considering the additional uncertainty deriving from random fluctuations of the offset, the total uncertainty on the actual magnetic field at end of an integration interval can be expressed as:

(23) $$\sigma \left(B\right)=\sqrt{3\frac{U_{\mathrm{RMS}}^{2}}{A_{c}^{2}}\sigma^{2}\left(t^{*}\right)+{\left(\frac{d\bar{U}}{dt}\right)}_{\mathrm{RMS}}^{2}T^{*2}}.$$

From this expression it can be seen that in the end, under the assumptions made, short integration intervals do not impact negatively the final measurement uncertainty, since an higher uncertainty on the measured voltage offset will be compensated by a shorter time for drift to accumulate.

## 4. Case Study on ELENA and PSB at CERN

The model of Equation (23) and the effectiveness of the related correction were validated experimentally on the ELENA ring and the PSB at CERN, chosen as representative of extreme magnetic cycle durations, respectively very long (up to 120 s) or very short (1.2 s). To this aim, the uncertainty of time measurement, the variation of the voltage offset over time (RMS of offset time derivative), and the correction interval T^*^ that guarantees 1 μT of field stability were assessed. In particular:

1. for the uncertainty on time measurement, in Section 4.1, the test setup with the experimental results are presented;
2. the mean offset and the observed fluctuations of the offset are estimated in Section 4.2, by means of experimental data of field drift in ELENA and PSB;
3. for the correction results, in Section 4.3 the experimental data discussed in the two previous subsections is used in Equation (23) to calculate the correction interval $T^{*}$ with the target uncertainty of 1 μT.

### 4.1. Uncertainty on Time Measurement

#### 4.1.1. Test Setup

The test setup for the timing analysis consists of a teslameter Metrolab PT2026 with probe 1226, and a small permanent-magnet dipole. Their main metrological characteristics are highlighted in Table 1.

In the experimental test, the teslameter was connected to the PC via Ethernet. The error coming from frequency measurement, taken from the basis of the specification of the manual [17], is approximately 0.01 ppm in a field produced by a standard permanent magnet and it is therefore negligible. In the measurement of the magnetic field, the continuous mode of operation was selected owing its flexibility and efficiency to request the measurement in an adaptive way. Thus, a request was issued by the supervision software when the correction was needed according to the following procedure:

1. request the instrument to make the measurement;
2. wait for the task end (polling);
3. read the result.

For this purpose, three timestamps were taken: (i) before the measurement ($t_{0}$), when the request is first made; (ii) after the measurement ($t_{3}$); and (iii) after the data transmission ($t_{4}$).

**Figure 2:**
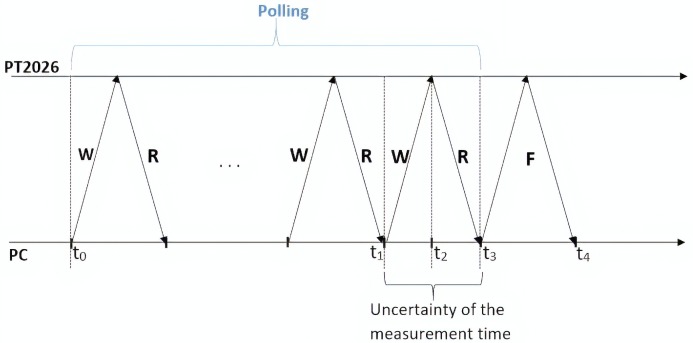
Communication diagram operation: write (*W*), read (*R*), and fetch *F*.

The operations of the communication over time are highlighted in Figure 2. The first request to the teslameter will generally have a negative result due to the polling time. After a number *n* of iterations, on average five, the bit *New Measurement Available* of the *Operation Status Register* will be set and the fetch phase will be performed to read the value. The average duration of a cycle, consisting of a write and a read, is about 7 ms.

The instant of time of the measurement is between the instant $t_{1}$ and $t_{3}$. It is assumed at the center of the interval [$t_{1}$,$t_{3}$] in order to minimize the maximum error:

(24) $$t_{2}=\frac{t_{3}+t_{1}}{2}\pm \underset{uncertainty}{\underset{︸}{\frac{t_{3}-t_{1}}{2}}}.$$

By means of these timestamp values, the measurement times and the readout delay were assessed. The overhead in addition to the measurement and transmission phases is less than 10 μs, thus turns out to be negligible. Therefore, the difference between $t_{2}$ and $t_{0}$ is assumed to represent the time needed for the measurement, while the difference between $t_{4}$ and $t_{2}$ represents the delay for the transmission.

The test was made with a set of 600,000 consecutive measurements and lasted about 10 h during the night.

#### 4.1.2. Experimental Results

**Figure 3:**
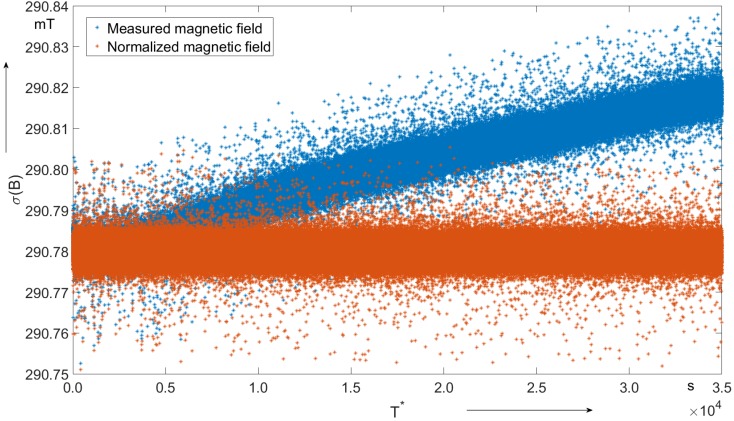
Measured and normalized magnetic field as a function of the time.

Figure 3 shows the measured and normalized magnetic field as a function of the time in blue and in orange respectively.

The blue graph shows a trend of about 1.16 nT/s, resulting in a field increase by 40.5 μT through the measurement. The relative drift is given by

(25) $$\frac{y_{f}-y_{i}}{y_{i}}=40.5\;\mathrm{ppm},$$

where $y_{f}$ and $y_{i}$ are the final and initial field value of the best fit, respectively. Using the temperature coefficient (Table 1), this drift corresponds to a thermal drift of 0.03 ${}^{\circ }$C (Equation (26)) during the night.

(26) $$-\frac{40.5}{1200}=-0.03\;{\;}^{\circ }C.$$

The statistics of the field values are given in Table 2.

The main aspects of the field analysis results are summarized by the following observations:

- with reference to Table 2, the relative dispersion of the field values is
  (27) $$\frac{\mathrm{Standard}\;\mathrm{Deviation}}{\mathrm{Mean}}=\frac{2\;\mu T}{290,780\;\mu T}=7\times 10^{-6}\approx 7\;\mathrm{ppm},$$
  fully compatible with the instrument specification (±5 ppm [17]);
- the mean field value is compatible with the nominal field of the magnet (Table 1).

For the timing analysis, the results of the statistical analysis of measurement time and readout delay are reported in Table 3.

The readout delay, on average, is less than the time for measurement, as expected.

### 4.2. Mean Offset and Observed Fluctuations of the Offset

**Figure 4:**
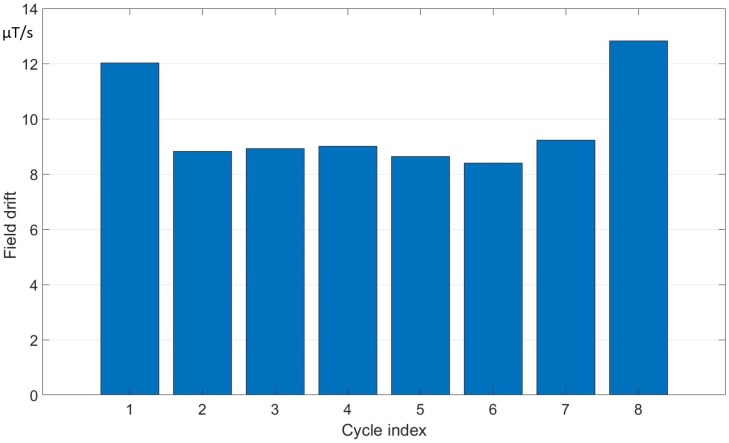
ELENA field drift measured over cycles of duration 30 s.

**Figure 5:**
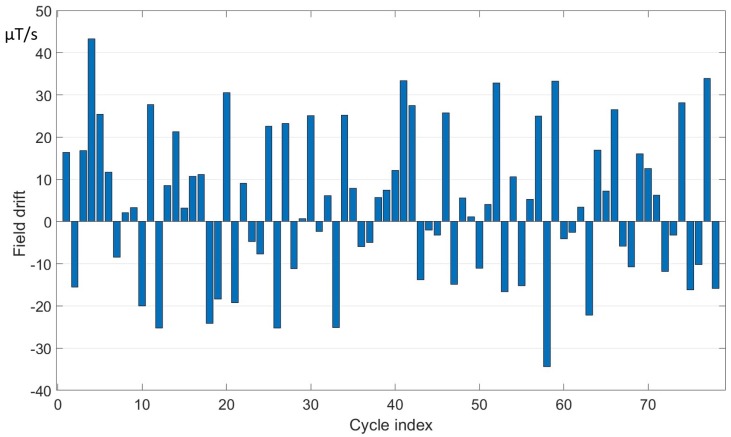
PSB field drift measured over cycles of duration 1.2 s.

Typical values of the field drift are estimated experimentally and they are different for the various machines. In Figure 4 and Figure 5, the results of field drift measurements are shown for ELENA and PSB, respectively.

The values of the field drift are plotted as a function of the cycle index. In the case of ELENA, a cycle lasted about 30 s, while, in the PSB, about 1.2 s (the maximum length of 120 s in ELENA is actually planned for a future operation period). The stability of the clocks of the integrator is in the order of few tens of ppm, while the stability of the power supply is within the ppm.

The mean offset and the observed fluctuations of the offset were calculated according to the Equations (8) and (9) respectively. The results are shown in Table 4.

For both machines, the average, the standard deviation, and the RMS value of the mean voltage offsets are reported in the third, fourth and fifth column, respectively. In the last two columns, the mean and standard deviation of the field drift measured in the existing systems are shown. The measured values of voltage offset and drift are up to about 45μV and 20 μT/s. While in ELENA the offset tends to vary just by 10% over a total observation time of 240 s, the relative variation in PSB is much larger; the sign of the effect changes and the average is much smaller than the standard deviation. These differences in behavior are consistent with the experience of similar systems at CERN and may be ascribed to a number of factors: the length of the cabling between the sensors and the acquisition system, and the level of electrical noise in the environment.

### 4.3. Correction Results

**Figure 6:**
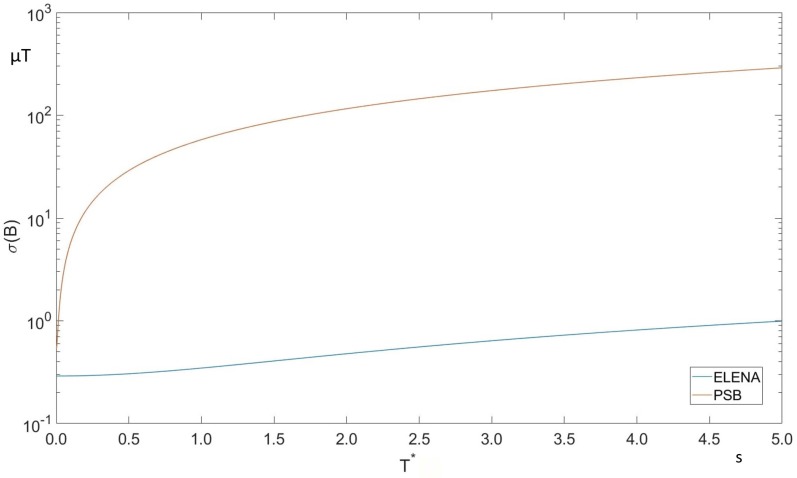
Field uncertainty after drift correction for PSB (orange) and ELENA (blue).

Based on the mathematical modeling of the drift and of the results obtained experimentally in ELENA and the PSB, combined with the results of the metrological characterization for the NMR, we have attempted to predict the value of the uncertainty of the field at the end of the next interval, through Equation (23), using the new feed-forward correction algorithm and the experimental results. This uncertainty grows linearly, as expected, with the length of the correction interval as shown in Figure 6. The values of the uncertainty of the existing system for the both machines and in case of the new feed-forward correction are given in Table 5.

The target tolerance of 1 μT can be reached by adopting a $T^{*}\leq 5$ s in ELENA (corresponding to 24 corrections per cycle) or $T^{*}\leq 15$ ms in the PSB (80 corrections per cycle). When compared to the RMS uncertainty of the existing measurement, this translates to a potential improvement of a factor about 20 in the PSB, but by as much as three orders of magnitude in ELENA. This big difference is due to the much longer plateaus of the ELENA cycle and confirms the validity of the choice of the feed-forward algorithm.

These values of $T^{*}$ should be compared with the duration that minimizes the uncertainty on the offset, as derived from Equation (20), i.e., 1.6 s for ELENA and 113 ms for the PSB. For ELENA, the acceptable $T^{*}$ is longer, meaning that accuracy of offset estimation is the dominant factor as far as field accuracy is concerned. For the PSB, conversely, the acceptable $T^{*}$ is much shorter, which means that the dominant field error source are the rapid fluctuations of the offset.

## 5. Conclusions

A feed-forward method to correct voltage integrator drift on long, quasi-static magnetic field plateaus has been presented and developed in detail. The typical voltage offsets observed in two of the real-time magnetic measurement systems currently in operation at CERN have been characterized first, including their time variability. The experimental time data taken from the permanent magnet test have been used to estimate the uncertainty on time measurement. In fact, the timing of the NMR teslameter at the heart of the new correction method has been determined, finding a 17.0 ms readout time jitter, mainly linked to the software triggering, which is so far the main limiting factor. In spite of this, the method shows great promise, in particular it is expected to meet the very strict specification of 1 μT field stability on ELENA plateaus as long as 120 s. This result represents an improvement of three orders of magnitude over the current state of the art, which warrants implementation of the method in the new B-train systems.

Unlike all other drift-reduction methods reported in the literature, the proposed method to estimate the voltage offset based on the measured field takes into account simultaneously all of its physical sources, both external and internal to the integrator. The cost is an increased system complexity and the need for a further independent instrument.

In the CERN context, this cost is largely outweighed by the benefits, which include the possibility to forego completely the forced insertion of dedicated zero cycles in the machine schedules. Future work includes (i) removing the restriction of quasi-static plateaus by means of different kinds of teslameter, trading off measurement accuracy with flexibility of utilization; (ii) a more consistent mathematical modeling of the time-changing offset as a stochastic process; and (iii) the extension of the real-time correction also to gain errors.

## Figures and Tables

**Table 1:** Features of the test setup.

Parameter	Value
Magnet type	Metrolab PM-1055050N
Magnet field	0.290 T @ 24 ${}^{\circ }$C
Magnet temperature coefficient	−1200 ppm/${}^{\circ }$C
Probe Model	1226
Probe Range	0.19–0.52 T
Teslameter Model	Metrolab PT2026
Teslameter S/N	00037
Teslameter accuracy	±5 ppm, independent of temperature
Teslameter measurement rate	up to 33 Hz

**Table 2:** Field measurement results.

Parameter	Value
	mT
Mean	290.780
Standard Deviation	0.002
Min	290.751
Max	290.805

**Table 3:** Results of real-time analysis of the teslameter.

Parameter	Measurement Time	Readout Delay	Total Time
	ms	ms	ms
Mean	38.5	5.9	44.3
RMS	42.1	7.2	47.5
Standard Deviation	17.2	4.3	17.0

**Table 4:** Voltage offset and field drift measured in ELENA and PSB.

Machine	$A_{c}$	$U_{\mathrm{avg}}$	$\sigma (\bar{U})$	${\left(\frac{d\bar{U}}{\mathrm{dt}}\right)}_{\mathrm{RMS}}$	$U_{\mathrm{RMS}}$	$\bar{\frac{{\mathrm{dB}}_{m}}{\mathrm{dt}}}$	$\sigma \left(\frac{{\mathrm{dB}}_{m}}{\mathrm{dt}}\right)$
	m${}^{2}$	μV	μV	μV/s	μV	μT/s	μT/s
ELENA	2.8	27.3	4.7	0.2	27.7	9.7	1.7
PSB	2.4	9.3	42.5	57.9	43.5	3.9	17.7

**Table 5:** Final results for a maximum cycle length Δt, including the maximum field drift (third column) and field error (fourth column) for the system as it is today, with no feed-forward correction. The acceptable T${}^{*}$ corresponds to σ(B)≤1μT in Figure 6.

Machine	Δt	$\frac{1}{A_{c}}U_{\mathrm{RMS}}$	$\frac{\Delta t}{A_{c}}U_{\mathrm{RMS}}$	Acceptable T${}^{*}$
	s	μT/s	μT	s
ELENA	120	9.9	1185.5	$T^{*}\leq 5$
PSB	1.2	18.1	21.7	$T^{*}\leq 0.015$

## Appendix A. Uncertainty of Fluxmetric Field Measurement

In this Appendix, the uncertainty of the field increment ΔB:

(A1) $$\Delta B=\frac{1}{A_{c}}\int_{t_{k}^{*}}^{t}V_{\mathrm{in}}\left(\tau \right)\;d\tau$$

computed in Equation (5) from voltage integration is assessed. The voltage is acquired by an ADC and the integral is computed numerically, according to:

(A2) $$\Delta B_{n}=\frac{\delta t}{A_{c}}\sum_{i=1}^{n}V_{\mathrm{in},i}$$

where δt is the sampling time and *i*, *n* are sample indices. By propagating uncertainties in Equation (A2), the uncertainty of the field increment can be expressed in relative terms as:

(A3) $$\frac{\sigma^{2}\left(\Delta B_{n}\right)}{\Delta B_{n}^{2}}=\frac{\sigma^{2}\left(A_{c}\right)}{A_{c}^{2}}+\frac{\sigma^{2}\left(\delta t\right)}{\delta t^{2}}+\frac{1}{n}\frac{\sigma^{2}\left(V_{\mathrm{in}}\right)}{V_{\mathrm{in}}^{2}}$$

The uncertainty component derived from the coil area, as discussed in Section 3.4, is of order $10^{-4}$ and represents the uncertainty on the geometrical scaling factor applied to the magnetic flux. The component associated to the sampling time reflects the jitter of the ADC’s digital clock, which is typically of order $10^{-6}$ and therefore negligible. The last component, associated with the sum of the voltage values, tends to vanish as the integration proceeds, due to random sampling errors canceling out quadratically on average. Considering, for example, a 16-bit ADC with a conservative noise level of 3 LSB, acquiring a weak 100mVRMS signal at 1MS/s, the relative uncertainty will drop below $10^{-6}$ after as little as 0.2 s. To conclude, for the long machine cycles main objective of the present work, the dominant source of uncertainty in the fluxmetric measurement is the effective surface of the induction coil:

(A4) $$\frac{\sigma^{2}\left(\Delta B\right)}{\Delta B^{2}}\approx \frac{\sigma^{2}\left(A_{c}\right)}{A_{c}^{2}}$$

The same reasoning would apply to any gain applied to the acquisition chain, such as the one of a preamplifier, that would appear as a multiplicative factor in Equation (A1).
